# Bacteroidetes to Firmicutes: captivity changes the gut microbiota composition and diversity in a social subterranean rodent

**DOI:** 10.1186/s42523-023-00231-1

**Published:** 2023-02-10

**Authors:** Hanna M. Bensch, Conny Tolf, Jonas Waldenström, Daniel Lundin, Markus Zöttl

**Affiliations:** 1 Department of Biology and Environmental Science, Centre for Ecology and Evolution in Microbial Model Systems (EEMIS), Linnaeus University, 391 82 Kalmar, Sweden; 2 Kalahari Research Centre, Kuruman River Reserve, Van Zylsrus, South Africa

**Keywords:** Captivity, Wild, Gut microbiota, Damaraland mole-rat, 16S amplicon sequencing

## Abstract

**Background:**

In mammals, the gut microbiota has important effects on the health of their hosts. Recent research highlights that animal populations that live in captivity often differ in microbiota diversity and composition from wild populations. However, the changes that may occur when animals move to captivity remain difficult to predict and factors generating such differences are poorly understood. Here we compare the bacterial gut microbiota of wild and captive Damaraland mole-rats (*Fukomys damarensis*) originating from a population in the southern Kalahari Desert to characterise the changes of the gut microbiota that occur from one generation to the next generation in a long-lived, social rodent species.

**Results:**

We found a clear divergence in the composition of the gut microbiota of captive and wild Damaraland mole-rats. Although the dominating higher-rank bacterial taxa were the same in the two groups, captive animals had an increased ratio of relative abundance of Firmicutes to Bacteroidetes compared to wild animals. The Amplicon Sequence Variants (ASVs) that were strongly associated with wild animals were commonly members of the same bacterial families as those strongly associated with captive animals. Captive animals had much higher ASV richness compared to wild-caught animals, explained by an increased richness within the Firmicutes.

**Conclusion:**

We found that the gut microbiota of captive hosts differs substantially from the gut microbiota composition of wild hosts. The largest differences between the two groups were found in shifts in relative abundances and diversity of Firmicutes and Bacteroidetes.

**Supplementary Information:**

The online version contains supplementary material available at 10.1186/s42523-023-00231-1.

## Introduction

The research field of the animal gut microbiota (the microbial community within the gut) is biased towards studies of traditional model organisms and domesticated laboratory animals [1, 2]. However, an increasing number of studies on wild animals have shown that the gut microbiota is highly variable within host species. The differences can for example be linked to changes in season, geographic location, diet, social group structures or individual host characteristics [3–8]. It is important to study the differences in gut microbiota composition between wild and captive animals to assess how they diverge and if knowledge based on one environmental condition can be applied to other conditions. Additionally, because the gut microbiota can be important to host health and fitness [9–11], these studies are also important for conservation programs and management of captive animals to preserve a natural gut microbiota [12, 13].

While dietary and seasonal changes are expected to explain a large proportion of variation in gut microbiota composition within and between wild and captive animals [7, 8, 14–17], other factors associated with captivity may also impact its composition [18, 19]. For example within social species, the gut microbiota is often shared and spread between group members, and group membership is therefore an important predictor of gut microbiota composition [4, 20, 21]. In captivity, social contact with conspecifics may be limited, or occur in a different way than between wild animals. This may alter the gut microbiota to become more divergent from the wild with time. Captive animals may also be housed outside their natural distribution which would decouple them from native seasonal patterns and natural sources of exposure of bacteria. Moreover, co-housing with other species of animals and exposure to humans may also alter the microbiota of captive animals. In a study on primates, for example, the gut microbiota alpha diversity decreased as the captive environment and conditions became more different from the wild [22]. Furthermore, specific but small changes can have large effects, for instance if the abundance of pathogenic bacteria differs between captive and wild environments [23]. Finally, energetic demands on the host and energy intake often vary between captivity and the wild, and can shape gut microbiota composition [24], so that an energetically less costly lifestyle in captivity may lower the host’s dependence on harbouring a specific microbial community within the gut.

The environment of captive animals is often less diverse than that of their wild conspecifics, which could cause a reduction of microbial diversity in the gut. However, the effect of captivity on gut microbiota alpha diversity can vary across taxa [18, 19], different captive populations [25], and a recent meta-analysis found no systematic effects of captivity on gut microbiota diversity [26]. For example, studies comparing diversity in gut microbiota of captive and wild animals have found that diversity in captive or domesticated animals can either be lower [e.g. 16, 22, 27–29, higher [e.g. 30, 31 or similar to that of wild animals [e.g. 23, 32. Together, this suggests that a higher diversity is not always the natural state and that the effect of captivity on gut microbiota diversity varies with host species and with environmental conditions in the captive environment [22]. In order to map effects of transition from the wild into captivity on gut microbiota composition, we need more studies comparing captive and wild populations, in particular of populations that have recently gone through this transition and where the composition of the gut microbiota of the ancestral population is known.

In this paper we describe and compare the bacterial gut microbiota (henceforth the microbiota) of wild and captive Damaraland mole-rats (*Fukomys damarensis*) originating from a population in the Southern Kalahari Desert using 16S amplicon sequencing of fecal samples. The captive group of animals has several unusual characteristics that make this comparison particularly interesting. It was established recently (animals were brought into captivity from the wild in 2013 and 2014) and because individuals can live longer than 10 years and start breeding at the age of two [33, 34], changes in the microbiota therefore likely reflect the changes that can be expected from one generation to the next. Unlike many other captive animals, the captive mole-rats are exposed daily to substrates directly taken from the original habitat so that a large part of the exposure to environmental microbes remains intact. In this study, we answer a number of questions regarding differences in bacterial gut microbiota composition and diversity between wild and captive mole-rats. First, we ask if wild and captive individuals differ in their gut microbiota composition. Second, we ask what taxa drive differences between the groups by testing for differentially abundant Amplicon Sequence Variants (ASVs) between the two groups and by investigating the ASVs driving the variation in the directions of the two groups in microbiota community composition. Third, we ask if alpha diversity is different between samples from wild and captive animals. Finally, we investigate the proportion of taxonomically unassigned taxa within the two groups to test the hypothesis that the captive individuals’ microbiota to a larger extent consists of taxa known from studies of gut microbiota of other, well-studied, host species.

## Methods

### The study species

The Damaraland mole-rat is a social subterranean rodent that lives in cooperatively breeding family groups (mean family group size 8.7)  which can breed in captivity in artificial tunnel systems or can be studied in the wild by trapping individuals in their natural burrow systems [35–38]. Despite being a relatively small rodent (adult body mass 90 to 200 g), Damaraland mole-rats can reach ages of more than 10 years in the wild and likely more than 15 years in captivity [34]. They are strictly herbivorous and feed on geophytes with their diet often dominated by a single species, the gemsbok cucumber (*Acanthosicyos naudinianus*) [39]. The tubers of these cucumbers are high in fibres, but low in protein and starch content and contain the animals’ entire requirement for water [35, 40]. To locate the tubers and expand the tunnel system the animals dig with their large frontal teeth and push up the sand to the surface. Energy requirements of digging behaviour is high, and has been estimated for captive Damaraland mole-rats to about five times that of resting metabolic rate [41]. To gain sufficient amount of energy from the tubers, the Damaraland mole-rats are believed to efficiently ferment fibres in their guts [39], which in turn suggests that a healthy and stable gut microbiota of wild Damaraland mole-rat is crucial for the host’s health and fitness.

### Sample and data collection

The samples used in this study were collected from 53 captive and 59 wild non-breeding Damaraland mole-rats. The individuals (51 females and 58 males) were commonly unrelated and originated from multiple social family groups (14 wild, 20 captive). The wild animals were captured as a part of a long-term population study of the Damaraland mole-rat population at the Kalahari Research Centre (− 26.977439, 21.832659), South Africa, and the captive animals were from the laboratory facility at the Kalahari Research Centre within the reserve. The captive part of the population was founded by wild caught individuals captured around the Kalahari Research Centre in 2013 (− 26.938854, 21.691686; − 26.890933, 22.079785; − 27.112075, 22.061217) and all but three of the sampled captive individuals were F1 and F2 generation from wild-caught individuals. All individuals were pit-tagged to allow individual identification.

The wild individuals were housed in a separate laboratory from the captive animals during captures until release back to their burrow system in the wild after a maximum of 7 days in the laboratory. Careful measures were taken to avoid transmission of bacteria between the two groups and contamination of samples. The captive Damaraland mole-rats were fed with sweet potato (*Ipomoea batatas*) while wild animals were provided their natural diet during captures and while temporarily housed inside the laboratory. In contrast to the natural diet in the wild, sweet potato is richer in starches and protein but poorer in fibres [40]. Captive animals in this study were provided daily with sand from the nearby area to promote digging behaviour and, while living in captivity, remained exposed to the soil microbes of their natural habitat.

The fecal samples were collected by placing animals inside a sterilised plastic box provided with paper and a small piece of food. The animals were checked frequently until defecation. Subsequently, the animals were released back to their family group members. For wild-caught animals, the fecal samples were the first fecal pellets after capture. Samples were collected and placed into a 1.5 ml sterile tube and then stored in a minus 80 °C freezer on site until transported on dry ice to the laboratory at Linnaeus University, Kalmar, Sweden.

### Library preparation and sequencing

The 16S library preparation, sequencing protocol and bioinformatic pipeline used in this study has previously been described in Bensch et al. [42] where detailed information on the workflow and pipeline can be found. Fecal samples of captive and wild Damaraland mole-rats were randomised on three 96-well plates, and for this analysis we used 56 samples from wild caught animals collected within the time range of the 53 samples from the captive animals, between the 6th of September and 9th of November 2019. On each plate we included four negative control samples by excluding the sample added in the first step of extraction and one mock community standard (25 μl ZymoBIOMICS Microbial Community DNA Standard) and DNA was extracted using the DNeasy PowerSoil Pro Kit (Qiagen). We amplified the DNA using the primers 341F (5′-CCTACGGGNGGCWGCAG-3′) and 805R (5′-GACTACHVGGGTATCTAATCC-3′) targeting the hypervariable V3-V4 region of the 16S rRNA gene and including adapter sequences for Illumina n5/n7 index primers [43, 44] using 25 μl reactions. PCR products were purified using AMPure XP magnetic beads and were used as templates for a second PCR adding a unique combination of Illumina n5/n7 index primers to each sample using 50 μl reactions. PCR-products were purified, DNA concentrations were determined using a Qubit fluorometer (Thermo Fisher Scientific) and equimolar amounts of each sample library were pooled together per 96-well plate into pools with final concentration 4 ng/μl. Pools were ﻿300-bp paired end sequenced following standard Illumina sequencing protocols on an Illumina MiSeq platform at the Swedish National Genomics Infrastructure (NGI) at SciLifeLab in Uppsala, Sweden.

### Bioinformatics and sequencing filtering

For bioinformatics, we followed Bensch et al. [42] and processed the raw reads from FastQ inputs using the Ampliseq workflow v1.2.0dev (https://nf-co.re/ampliseq/1.2.0, [45]) which uses Cutadapt v.2.8 [46] and the implementation of DADA2 v.1.10.0 [47] in QIIME2 v2019.10.0 [48] to create ASVs tables. Quality of the sample reads was checked with FastQC v0.11.8 [49] and MultiQC v1.9 [50], and taxonomy was assigned against the SILVA database v.132 [51].

### Quality check and filtering of NGS data

All analyses post Ampliseq were conducted in R version 4.1.2 [52], using functions within the packages tidyverse, vegan and phyloseq [53–55]. To increase the number of reads per sample, we combined reads of samples on plates that had been sequenced twice (plate 2 and 3).

We filtered away 210 ASVs identified as contaminants by the decontam-package v1.8.0, using a threshold of 0.5 and plate number as batch argument [56], identifying a total 8,709,371 reads and 5,042 unique ASVs in the 109 fecal samples from 53 captive and 56 wild Damaraland mole-rats. Mean number of sequences per sample was 79,902 (SD = 40,183), and samples from wild animals had significantly larger library sizes than samples from captive animals (mean captive = 65,054 ± 33,006 SD, mean wild = 93,955 ± 41,559 SD, LMM *p* = < 0.001, Additional file 1: Table S1).

### Statistical analysis and measures of diversity

To test for differences in beta diversity of the bacterial community of the microbiota between wild and captive animals, we performed a principal component analysis (PCA) on centred log ratio (CLR) transformed counts of ASVs using the rda function in the vegan package [54]. We performed a Permutational Multivariate Analyses of Variance (PERMANOVA) with the adonis2 function in vegan [54] on a Euclidean distance matrix of CLR-transformed counts with group (wild or captive) and library size as factors with plate number as strata argument to explore the marginal effects explained by study group origin (wild or captive). To test for differences in dispersion in beta diversity between the two groups, we ran multivariate homogeneity of groups dispersion test with betadisper function in vegan [54].

We explored the taxa driving the microbiota community composition in the directions of the two groups by focusing on the ASVs with the 2% highest and lowest loading scores on the first PC axis (N = 101 unique ASVs in each direction) which clearly separated the two groups. Further, we tested for differentially abundant ASVs between the two groups with an Analysis of Compositions of Microbiomes with Bias Correction (ANCOM-BC) with the ANCOMBC package [57], correcting for multiple testing with Benjamini–Houchberg false discovery rate correction [58].

Sample ASV richness was estimated with the breakaway package [59] which provides standard errors and correction for incomplete sampling. This method uses the complete dataset without rarefying, which has been common practice in bacterial alpha diversity estimates of microbiota data but can result in the false impression of unequal richness [60]. We used the betta_random function [59] to test the hypothesis that alpha diversity did not differ between the two groups, modelling the estimated ASV richness as a response of the fixed factor group (captive or wild) while controlling for variation between sequencing plates using plate number as a random factor and taking the uncertainty and error of the diversity estimate into account with the ses-argument. To explore the taxa where ASV richness differed between the two groups, we counted the number of unique ASVs within each sample group of the most common phyla. We tested for differences in the number of unique ASVs per phylum between the two groups with Wilcox signed rank tests as the number of ASVs were non-randomly distributed for most phyla, using Bonferroni correction of *p* values for multiple testing (*p*_*adj*_).

We tested for differences in library sizes and the ratio of relative abundances of Firmicutes/Bacteroidetes (F/B-ratio) between the two groups with linear mixed models using the lme4 package [61], with plate number as random factor and group as fixed factor. Difference in body mass index (fatness) between captive and wild animals was tested with a linear model fitting group as fixed factor. For all statistical tests, we defined *p* < 0.05 as threshold of statistical significance.

## Results

### Difference in gut microbiota composition

**Fig. 1:**
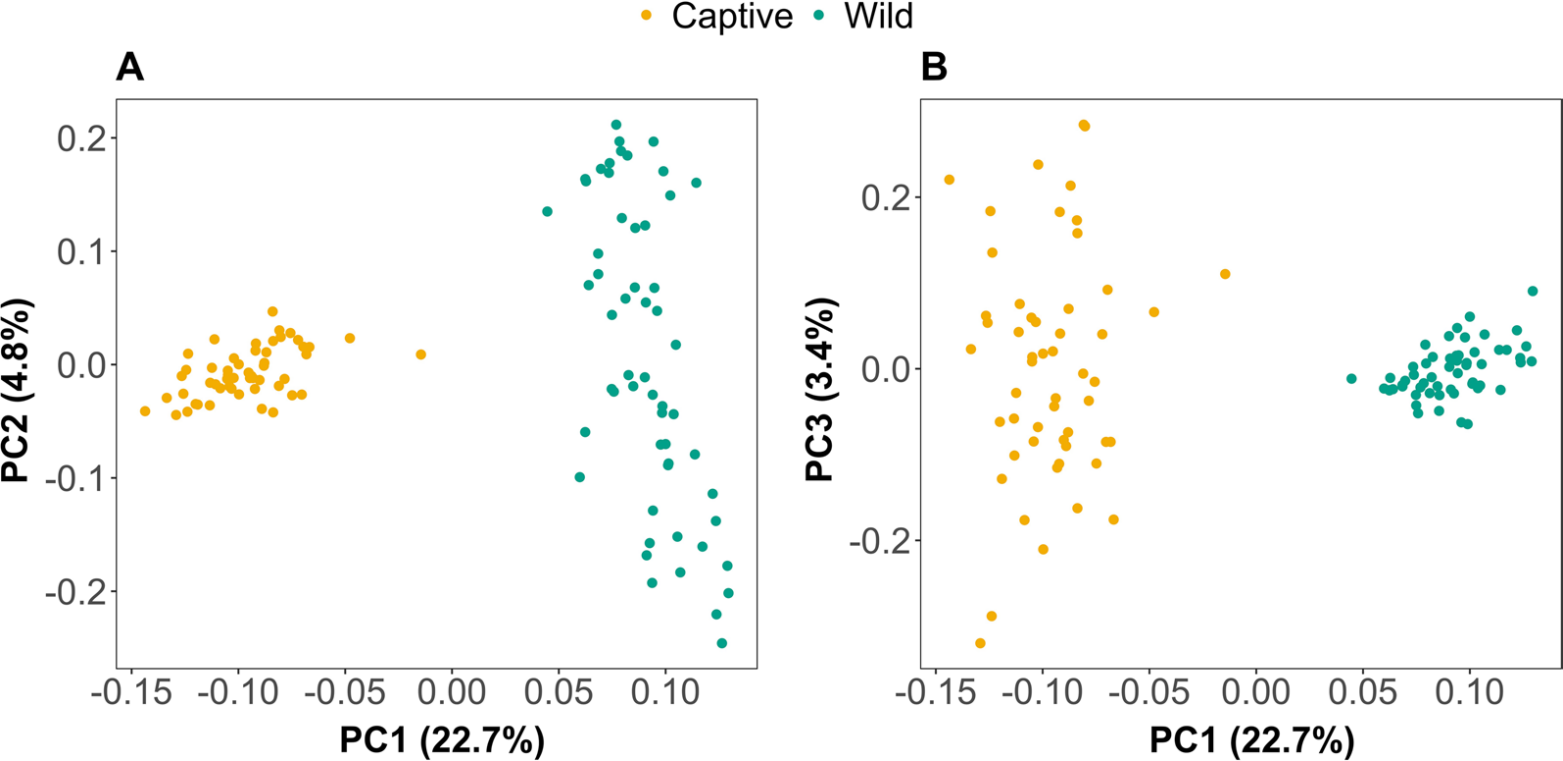
Between-sample dissimilarity of fecal samples from captive and wild-caught Damaraland mole-rats based on Euclidean distances. **A** First and second principal component (PC1 & PC2), and **B** first and third principal component (PC1 & PC3). Each point represents a sample (N_captive_ = 53, N_wild_ = 56) and are coloured according to origin of the sample (yellow = captive; blue-green = wild)

Samples from wild and captive Damaraland mole-rats were clearly separated by the first principal component in our PCA using Euclidean distances based on CLR-transformed counts. Group (wild or captive) described almost 19% of the total variation in microbiota community composition (PERMANOVA: *p* < 0.001, F = 25.8183, R^2^ = 0.18676, Fig. 1). There were however significant differences in beta dispersion between the two groups (*p* < 0.001, F = 50.729), and variation on the second principal component (PC2) separated samples from wild animals (Fig. 1a), while variation on the third principal component separated samples from the captive animals (Fig. 1b).

**Fig. 2:**
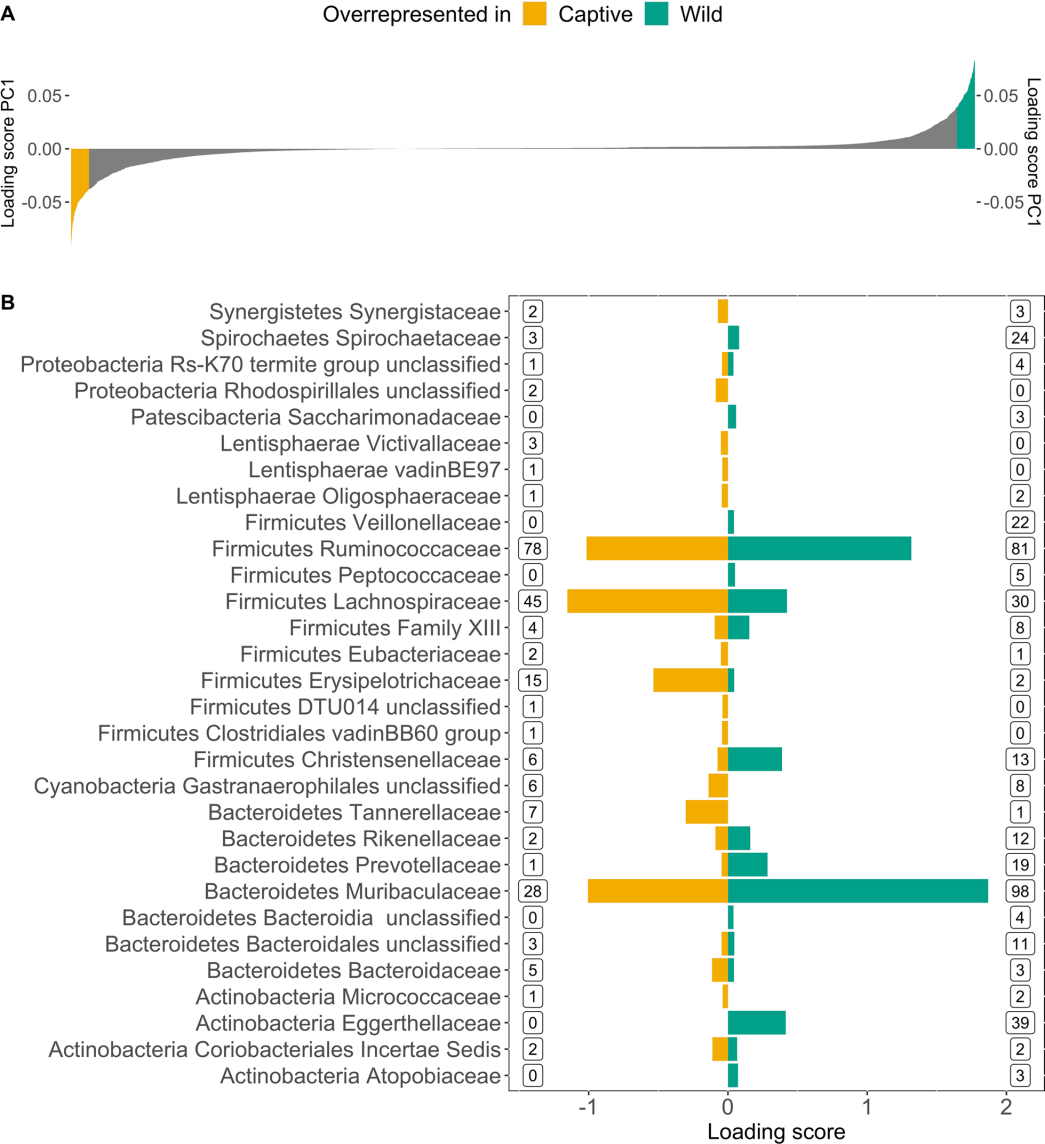
Taxa driving the separation of samples along the PC1 axis. **A** Loading scores of PC1 (Fig. 1) sorted for all ASVs on x-axis. ASVs with the top 2% loading scores (N = 101 ASVs) are filled in blue-green and correspond to ASVs characterising wild gut microbiota and ASVs filled in yellow correspond to ASVs with the 2% with the lowest loading scores (101 ASVs) characterising captive gut microbiota of Damaraland mole-rats. **B** Summed loading scores per family of ASVs within the 2% tails of loading scores of PC1 shown in plot A. The colour of the bars represents the group in which the ASVs were overrepresented, with negative loading scores associated with captive samples (yellow) and positive with wild samples (blue-green). The numbers within the boxes are the numbers of differentially abundant ASVs (N = 620 out of 690) identified with ANCOM within each of the families and their direction of driving the variation

**Fig. 3:**
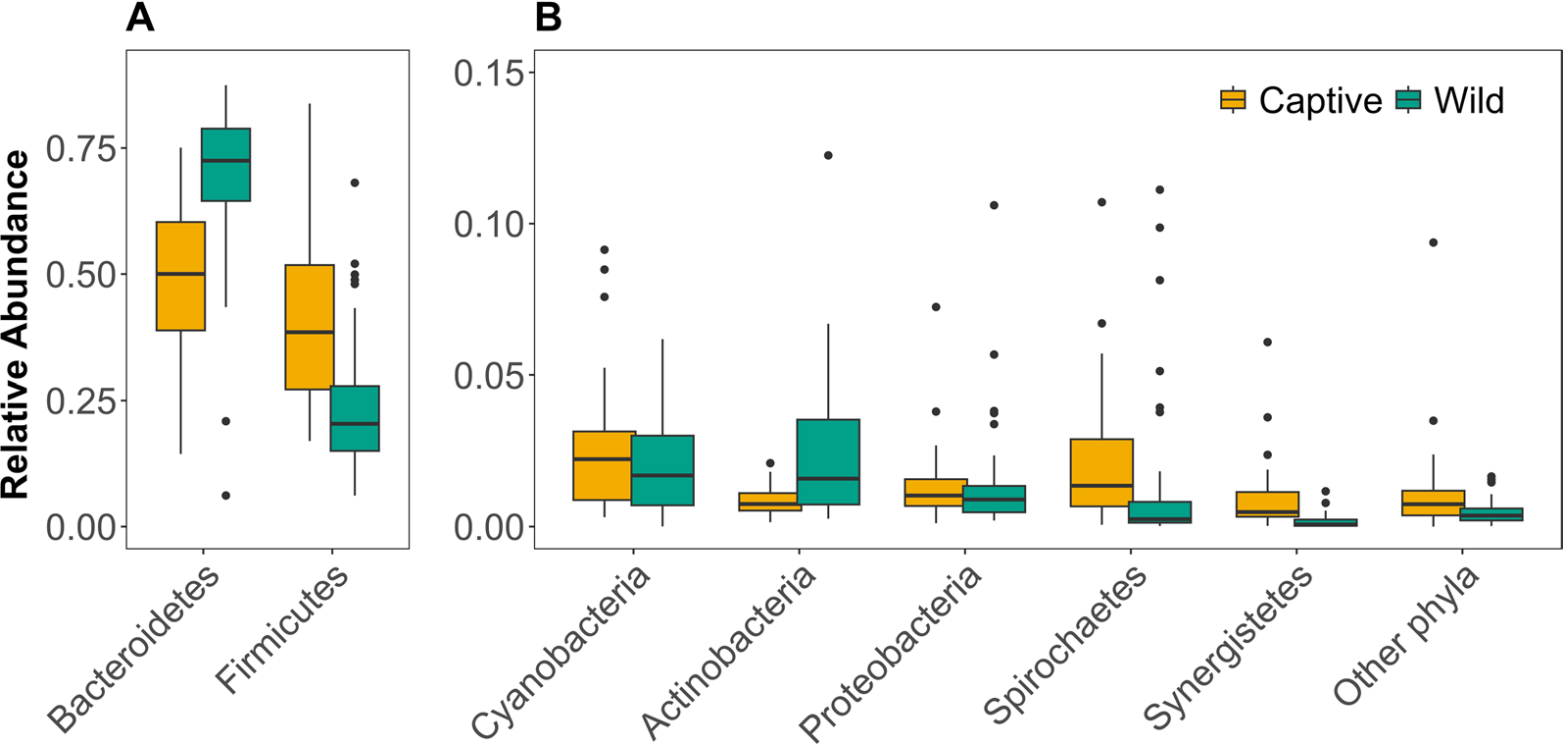
Boxplots of relative abundances for each sample and the seven phyla with 100% prevalence within wild and captive faecal samples from the Damaraland mole-rat. **A** The two dominating phyla, Firmicutes and Bacteroidetes. **B** Cyanobacteria, Actinobacteria, Proteobacteria, Spirochaetes and Synergistetes and other phyla, representing the sum of relative abundance of phyla not represented in all samples. Yellow boxes represent samples from captive individuals and blue-green boxes samples from wild individuals. Note the different y-axis scaling in the two panels

A total of 2816 ASVs were unique to the captive animals, and 1502 to the wild animals. The 724 ASVs that were shared between the two groups contained > 80% of the reads of the complete dataset and were dominated by Bacteroidetes and Firmicutes (Additional file 1: Table S2), just like the full dataset. When testing for differentially abundant ASVs between the wild and captive animals, we identified 690 ASVs of 59 families as differentially abundant, primarily from the Firmicutes and Bacteroidetes phyla (ANCOM, Additional file 1: Fig. S1, Additional file 2: Table S3). Of these, 225 ASVs had higher abundances in captive animals and 465 in wild animals (Additional file 1: Fig. S1, Additional file 2: Table S3). Out of the 690 differentially abundant ASVs, 620 were within the families represented in the 2% tails of PC1 (Fig. 2) and were dominated by the phyla Firmicutes and Bacteroidetes (Additional file 1: Fig. S2), the two most common phyla within both groups (Fig. 3). Samples from wild animals had higher relative abundances of Bacteroidetes while samples from captive animals had increased relative abundances of Firmicutes (Fig. 3a). Together this resulted in a difference of the Firmicutes/Bacteroidetes (F/B-ratio) between wild and captive individuals (Mean wild = 0.57 ± 1.47 SD, captive = 1.16 ± 1.16 SD, LMM *p* = 0.025, Additional file 1: Fig. S3a, Table S4). Despite the overall pattern, there were families within the phylum Bacteroides that were overrepresented in the captive group and families within Firmicutes that were overrepresented in the wild group (Fig. 2b). For example, the Firmicutes family Christensenellaceae, had heavier loads in the direction of wild samples, and the Bacteroidetes family Tannerellaceae toward captive samples (Fig. 2b). Among the less abundant phyla, ASVs and families within Actinobacteria and Spirochaetes weighed toward wild samples, while Lentisphaerae, Synergistetes and Cyanobacteria (ASVs belonged to a unclassified family of Gastranaerophilales, a non-photosynthetic bacterium belonging in a new candidate phylum of Cyanobacteria [62]) toward captive samples (Fig. 2b).

### Difference in alpha diversity

**Fig. 4:**
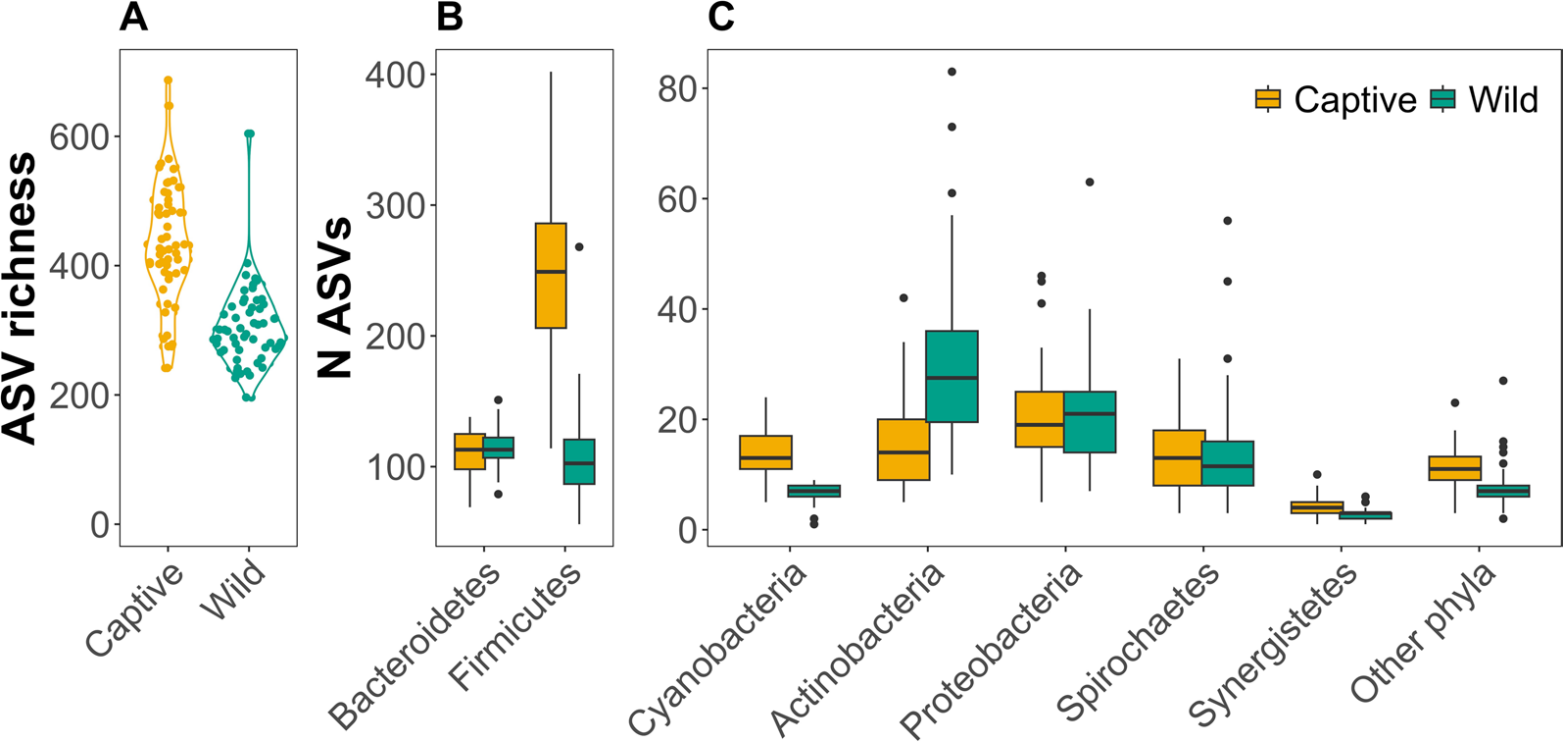
ASV richness of fecal samples from wild and captive Damaraland mole-rats. **A** Violin plots of ASV richness of wild and captive Damaraland mole-rats. Each point represents a sample (N_captive_ = 53, N_wild_ = 56). **B**, **C** Boxplots of number of unique ASVs for the seven phyla with 100% prevalence within wild and captive faecal samples from the Damaraland mole-rat. **A** The two dominating phyla, Firmicutes and Bacteroidetes. **B** Cyanobacteria, Actinobacteria, Proteobacteria, Spirochaetes and Synergistetes and a box with the including other remaining phyla, representing the sum of unique ASVs within phyla not represented in all samples. Yellow boxes represent samples from captive individuals and blue-green boxes samples from wild individuals. Note the different y-axis scaling in panels **B** and **C**

Captive animals had significantly higher estimated ASV richness than wild animals (mean captive = 436.205 ± 92.683 SD, mean wild = 305.067 ± 61.252 SD, *p* < 0.001, Fig. 4a, Additional file 1: Table S5). This difference was largely explained by much higher diversity and number of different ASVs of Firmicutes in captive samples (Fig. 4b, *p*_*adj*_ < 0.001, Additional file 1: Table S6). When looking deeper into the taxonomy within Firmicutes, the increase in richness of captive animals was largely due to an increased richness of the family Ruminococcaceae (Additional file 1: Fig. S4)*.* Notably, although relative abundance of Bacteroidetes was higher in wild samples (Fig. 3a), there was no difference in the number of unique ASVs of this phylum between wild and captive (*p*_*adj*_ = 1, Additional file 1: Table S6) and only Actinobacteria had significantly higher richness in wild samples compared to captive but incomparable to the magnitude of differences in Firmicutes (Fig. 4c).

### Taxonomically unclassified taxa

All ASVs were classified to domain, and the percentages of classified ASVs decreased further down the taxonomy with less than 10% of ASVs assigned at species-level (Additional file 1: Table S7). Notably, ASVs within the wild dataset had lower percentages of classified taxa than ASVs within the captive dataset within all taxonomic levels (Additional file 1: Table S7).

## Discussion

Similar to other studies comparing gut microbiota of wild and captive conspecifics [e.g. 16, 63–65], we found that the bacterial microbiota community in fecal samples from captive Damaraland mole-rats differed both in composition and diversity from wild animals. Our analysis identified 690 ASVs that were significantly differentially abundant between the two groups. These ASVs corresponded well with those responsible for driving the difference between the two groups in our PCA analysis and were dominated by the most common taxa, with ASVs within the phylum Bacteroidetes being overrepresented within samples from wild-caught animals while Firmicutes ASVs were overrepresented in captive individuals.

Similar to other mammals, the gut microbiota of both wild and captive Damaraland mole-rats were dominated by Bacteroidetes and Firmicutes [19], but the ratio of these was reversed between the two groups, with captive animals having a higher Firmicutes/Bacteroidetes ratio (F/B-ratio) compared to wild animals. Changes in gut microbiota composition and F/B-ratios of wild animals have previously been linked to changes in diets with seasonality [6, 8]. In a study on wild sifakas (*Propithecus verreauxi*) relative abundances of Firmicutes increased in the dry season while Bacteroidetes increased in the wet season when animals increased their intake of fruits [6]. These observations were similar to changes in relative abundances of Bacteroidetes in an extensive study on wild geladas (*Theropithecus gelada*) that during dry seasons with a fibre (and lignin) rich diet had a lower F/B-ratio compared to wet seasons [8]. Our data shows a different pattern with higher F/B-ratio in captive mole-rats, which had a starch-richer but fibre-poorer diet compared to the wild-caught animals. Instead, a similar pattern of changes in F/B-ratio to our study was found in a study on gut microbiota of brown bears (*Ursus arctos*) where the transition from an active lifestyle during summer to hibernation in winter was associated with an increase of Bacteroidetes and a decrease of Firmicutes [17]. These changes were suggested to be associated with caloric restriction [17]. Notably, captive animals in our study are significantly fatter than wild animals (Additional file 1: Fig. S3b), and it is possible that the more energetically costly lifestyle of wild Damaraland mole-rats is partly responsible for the change of the gut microbiota composition of captive animals. An increased F/B-ratio has been associated with obesity and gut dysbiosis in humans and mice [66, 67]. However, more recent data, including a meta-analysis, failed to detect any association between this ratio and obesity [68–70]. In light of this, the putative association between the F/B-ratio, caloric restriction and body mass index of hosts in our system, would require further investigations to unravel explanations and mechanisms. Because our study was not designed to control for all factors that could potentially affect the gut microbiota composition in captivity, disentangling the influence of different factors within the captive environment on the gut microbiota composition is difficult.

We did not detect any specific bacterial families that were overrepresented among ASVs that drove the gut microbiota of the captive animals away from the wild animals. Instead, ASVs strongly associated with wild animals were commonly classified as members of the same bacterial families as those strongly associated with captive animals. For example, the Firmicutes family Ruminococcaceae which contain bacteria that are efficient fermenters of fibre [71] and had numerous ASVs overrepresented in both groups with similar loadings in both directions on the PC1. Another important fibre fermenter family within Firmicutes, Lachnospiraceae [71], had on the other hand more ASVs overrepresented in captive than wild samples. These families were also represented by different ASVs driving variation on gut microbiota composition in the directions of both wet and dry seasons in the gelada gut microbiota [8]. The only Firmicutes family in our study that clearly weighed heavier toward wild samples was Christensenellaceae, a family that has been suggested to be associated with human health and fibre fermentation [72, 73]. Within Bacteroidetes there were bacterial families that are known to contain fibre-degrading bacteria too, such as the families Prevotellaceae and Bacteroidaceae [74]. Although not responsible for driving as much variation as some of the more highly abundant families within Firmicutes and Bacteroidetes, ASVs of the family Spirochaetaceae were overrepresented in wild samples. This family was dominated by the genus *Treponema*, a genus suggested important for fibre digestion in wild naked mole-rats (*Heterocephalus glaber*) [75]. It is possible that the overrepresentation of this taxon in wild Damaraland mole-rats relates to the fibre rich diet of the wild animals, though other factors that vary between the captive and wild environment may also contribute to this difference.

In contrast to some other studies on captive mammals, we found that captive Damaraland mole-rats had higher alpha gut microbiota diversity than their wild-caught conspecifics. Although there are other known exceptions [19, 26], captive mammals often show lower gut microbiota diversity than wild [16, 27–29]. When other wild-caught rodents were brought into captivity their gut microbiota diversity decreased [76], which was also true for another subterranean species, the solitary blind mole-rats (*Spalax leucodon*) [77]. However, the diet of the blind mole-rats in the wild was unknown [77] and decreases in gut microbiota diversity may be related by change to a more uniform diet. By contrast, the diet of wild Damaraland mole-rats is likely dominated by a single species of tuber throughout the year [35], and a decrease in gut microbiota diversity in captivity in this species due to a decrease in diet diversity would therefore not be expected. Instead, captivity likely exposes animals to a novel set of sources of bacterial transmission that can alter and increase their gut microbiota composition and diversity, while the maintained daily contact to environmental bacteria by adding sand as substrate may facilitate the maintenance of some of the environmentally induced gut microbiota. For example, the captive animals in our study had been introduced to a new diet. Another reason for increased gut microbiota diversity among captive animals could be that social groups are housed within artificial tunnel systems but are regularly taken out for husbandry and transmission between groups is likely much more common than in the wild which adds an additional source of bacterial exposure. Lastly, animals in captivity were regularly handled by humans which may also transmit bacteria that alters and replaces the wild microbiota, as for example in captive primates [22, 65]. This corresponds with the fact that ASVs within our captive dataset were typically assigned taxonomy more specifically than the captive dataset. This may indicate that a larger fraction of the bacteria within the samples from captive animals were associated with humans or other sources where we currently have more sequence information on bacterial taxonomy.

When investigating what taxa explained the higher diversity within gut microbiota of captive animals, we found that this was driven by increased number of Firmicutes ASVs compared to wild animals. This corresponded well to the increased relative abundance of Firmicutes in captivity, but we did not detect any difference in ASV richness within Bacteroidetes between wild and captive individuals although wild animals had much higher relative abundance of this phylum than captive animals. Interestingly, the Firmicutes family that was responsible for most of the increased richness in captive animals, Ruminococcaceae, did not have higher relative abundances in captive animals. Increased richness of a bacterial taxon clearly does not predict changes in abundance, calling attention to the complexity of the gut microbiota community and compositional nature of 16S data. So far although captive and wild hosts often differ in gut microbiota diversity [19, 26], few studies have looked into what taxonomic groups are responsible for changes in alpha diversity between captive and wild hosts. For example, in stark contrast to our study, a study on effects of captivity on gut microbiota of deer mice (*Peromyscus maniculatus*) showed that wild animals had higher diversity and higher ratio of relative abundance of Frimicutes/Bacteroidetes than captive animals [29]. What taxa increased in richness in wild hosts was however not reported, and it would be interesting to investigate if shared taxa are responsible for differences in alpha diversity between populations and environmental conditions across different study species. Together this also highlights the open question whether and how the relative abundance and ASV richness of a specific taxon affect the host.

## Conclusion

In this study, we found clear differences between the gut microbiotas of wild and captive Damaraland mole-rats, thereby adding to the growing list of studies exploring effects of captivity on gut microbiota composition. Captive animals harboured a bacterial community with higher F/B-ratios and the variation in beta diversity between captive and wild group was explained to a large proportion by ASVs within these two phyla. The ASVs driving the difference between the wild and captive individuals in either direction were commonly representatives of the same bacterial families. Given the large variation in relative abundances of the two dominating phyla between wild and captive animals it is possible these differences reflect important differences for host digestion efficiency, as animals were fed sweet potato in captivity instead of the natural diet of gemsbok cucumber.

Our study also shows that the gut microbiota alpha diversity of captive animals can be drastically increased compared to wild conspecifics. It has been proposed that a more diverse microbial community has higher resilience and stability [78], which in turn can play a key role for host health as diverse and stable microbiotas can outcompete pathogens better [79]. Moreover, our study suggests that a higher diversity is not always the natural state, and that the gut microbiota diversity can differ in both directions between wild and captive groups. Instead, correlates of gut microbiota diversity with host health should perhaps be considered in relation to other individuals from the same environment and conclusions on health status of an individual should not be drawn on alpha diversity alone. Lastly, the higher proportion of unassigned taxa in our wild dataset suggests a continued bias of microbiota studies of captive and laboratory systems and a future need of studies on gut microbiota of wild animals. Importantly, the gut microbiota is a complex community, and in this paper we focused on its bacterial composition. Future research is consequently needed on the other microorganisms in this environment such as fungi and viruses for a more complete understanding of its complexity and function.

## Supplementary Information

**Additional file 1.** Supplementary Tables and Figures.

**Additional file 2.** Supplementary Table S3.

## Data Availability

The raw 16S sequences are available at NCBI short read archive (SRA): PRJNA781121 and the BioSample numbers of samples used in this study can be found in Supplementary material Additional file 1: Table S8. The R scripts code used are available at https://github.com/HannaBensch/WildvsCapDMR.
