# Supplementary material for: Bacteroidetes to Firmicutes: captivity changes the gut microbiota composition and diversity in a social subterranean rodent
Source: Anim Microbiome. 2023 Feb 10;5:9. doi: 10.1186/s42523-023-00231-1 (PMC9912604; doi:10.1186/s42523-023-00231-1)
Supplement: Supplementary file 1 — Additional file 1. Supplementary Tables and Figures. [file 42523_2023_231_MOESM1_ESM.docx]

**Supplementary material “*Bacteroidetes to Firmicutes - captivity changes the gut microbiota composition and diversity in a social subterranean rodent*”**

**Table S1.** Summary linear mixed model (LMM) testing differences in library size between the wild and captive group of samples.

|  | **NbReads** | |
| --- | --- | --- |
| *Predictors* | *Estimates* | *p* |
| (Intercept) | 64667.40 (43864.76 – 85470.05) | **< 0.001** |
| Group [Wild] | 29594.58 (16089.41 – 43099.75) | **< 0.001** |
| **Random Effects** | | |
| σ^2^ | 1247482815.08 | |
| τ_00_ _Plate_No_ | 258687339.04 | |
| ICC | 0.17 | |
| N _Plate_No_ | 3 | |
| Observations | 109 | |
| Marginal R^2^ / Conditional R^2^ | 0.128 / 0.278 | |

**Table S2.** The taxonomic assignment of the 724 ASVs shared between wild and captive samples at phylum level. Number of ASVs, sum of reads and proportion of reads of complete data set summarized. The reads of these ASVs sum up to > 80% of the complete dataset.

| **Phylum** | **NASVs** | **Sum of Reads** | **Prop reads full dataset** |
| --- | --- | --- | --- |
| Bacteroidetes | 241 | 4461346 | 0.512 |
| Firmicutes | 223 | 1999126 | 0.23 |
| Cyanobacteria | 18 | 157829 | 0.018 |
| Actinobacteria | 52 | 116910 | 0.013 |
| Proteobacteria | 90 | 117487 | 0.013 |
| Spirochaetes | 64 | 107864 | 0.012 |
| Synergistetes | 10 | 30469 | 0.003 |
| Other phyla | 26 | 33106 | 0.004 |


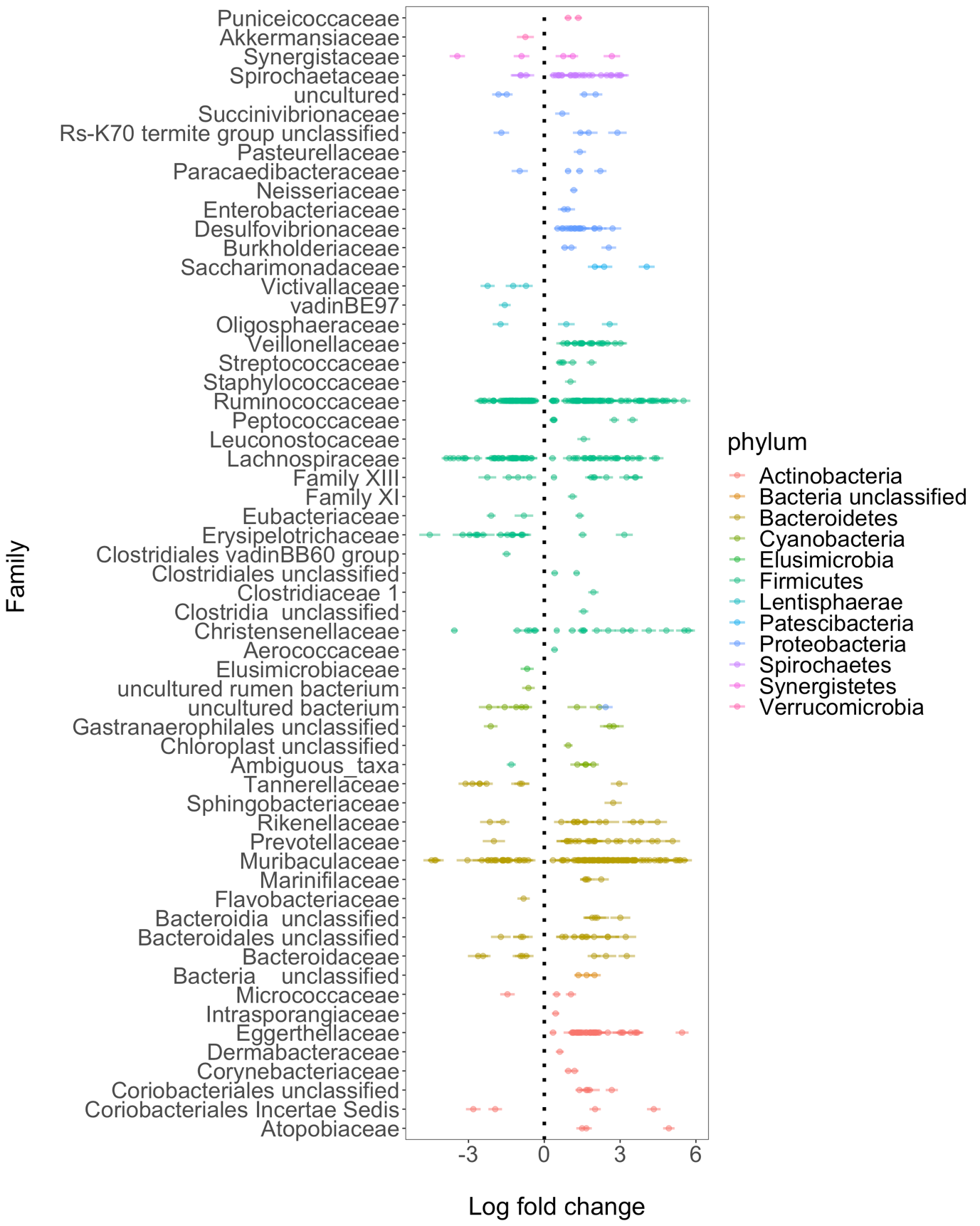


**Figure S1.** Differently abundant ASVs in the gut microbiota of captive and wild Damaraland mole-rats grouped by family on y-axis. Points and bars represent the log-fold change (effect size) of the 680 unique ASVs with p_adj_ < 0.05 and the 95% confidence intervals derived from the ANCOMBC model, with negative values indicating higher abundance in captive samples and positive values indicating higher abundance in wild samples. Points are coloured and sorted according to phylum, described in the legend in the figure.


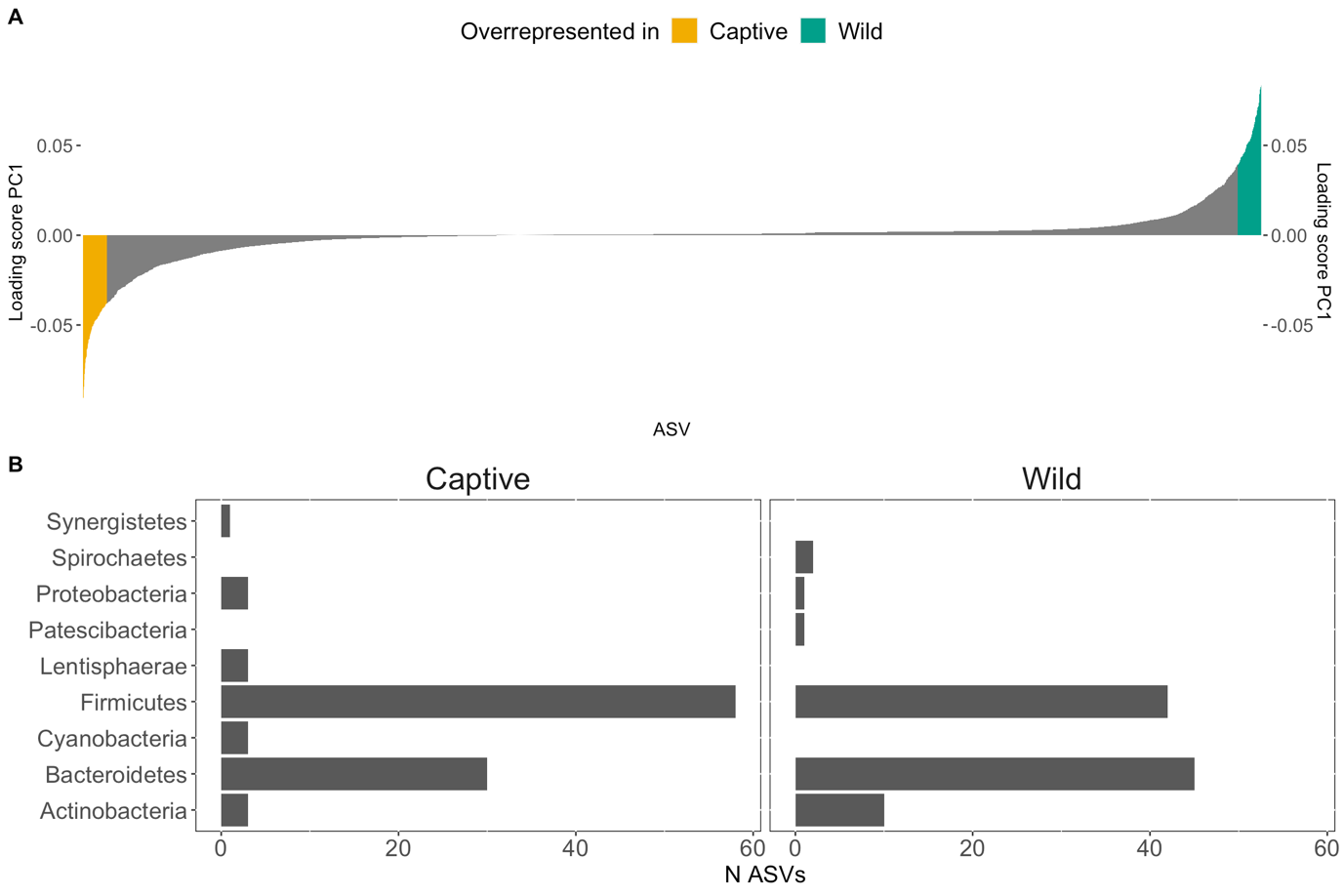
**Figure S2.** Taxa separation of samples along the PC1 axis. A) Loading scores of PC1 (Fig. 1) sorted for all ASVs on x-axis. ASVs with the top 2% loading scores (N = 101 ASVs) are filled in blue-green and correspond to ASVs characterising wild gut microbiota and ASVs filled in yellow correspond to ASVs with the 2% with the lowest loading scores (101 ASVs) characterising captive gut microbiota of Damaraland mole-rats. B) Sum of unique ASVs belonging to each phylum as either associated with “captive" or "wild" in plot A.

**Table S3.** ﻿Results from ANCOM, table with amplicon sequencing variants (ASVs) significantly differentially abundant (*p*-adj < 0.05) in the gut microbiotas of wild and captive Damaraland mole-rat.

*Added as separate supplementary table:* ***TableS3.tsv***

**Table S4.** Relative abundances Firmicutes/Bacteroidetes (F/B-ratio) ratio Liner Mixed Model results.

|  | **log(F/B-ratio)** | | | | |  |  |
| --- | --- | --- | --- | --- | --- | --- | --- |
| *Predictors* | *Estimates* | *std. Error* | *CI* | *p* | |  |  |
| (Intercept) | -0.19 | 0.12 | -0.42 – 0.04 | | 0.104 | |  |
| Group [Wild] | -0.98 | 0.16 | -1.30 – -0.67 | | **<0.001** | | |
| **Random Effects** | | | | | |  |  |
| σ^2^ | 0.67 | | | | |  |  |
| τ_00_ _Plate_No_ | 0.00 | | | | |  |  |
| ICC | 0.00 | | | | |  |  |
| N _Plate_No_ | 3 | | | | |  |  |
| Observations | 109 | | | | |  |  |
| Marginal R^2^ / Conditional R^2^ | 0.267 / 0.270 | | | | |  |  |

**Table S5.** Model summary betta-random estimated richness with the breakaway package [1]. The intercept corresponds to mean richness for samples from captive animals.

|  | **Estimates** | **Standard Errors** | ***p*-value** |
| --- | --- | --- | --- |
| **Intercept** | 430.9377 | 7.316445 | 0 |
| **Group Wild** | -129.6338 | 10.180837 | 0 |

**Table S6.** Results Wilcoxon sum test to differences in the number of unique ASVs per phylum between wild and captive animals. Mean and sd number of unique ASVs per group (wild or captive) provided. *p*-adjust are *p*-values adjusted with Bonferroni adjustment for multiple testing.

| **phylum** | **Mean Captive** | **Mean Wild** | **Sd Captive** | **Sd Wild** | ***p-*value** | ***p*-adjust** |
| --- | --- | --- | --- | --- | --- | --- |
| Bacteroidetes | 111.5 | 114.7 | 17.6 | 14.3 | 0.776 | 1 |
| Firmicutes | 245.3 | 106.5 | 66.1 | 32.8 | <0.001 | <0.001 |
| Cyanobacteria | 13.6 | 6.7 | 4.2 | 1.9 | <0.001 | <0.001 |
| Actinobacteria | 14.9 | 30.4 | 7.8 | 15.3 | <0.001 | <0.001 |
| Proteobacteria | 20.8 | 21.3 | 8.7 | 9.5 | 0.75 | 1 |
| Spirochaetes | 13.8 | 14 | 7.2 | 9.9 | 0.631 | 1 |
| Synergistetes | 4.4 | 2.7 | 1.8 | 1.3 | <0.001 | <0.001 |
| Other phyla | 11.2 | 7.6 | 4.1 | 3.8 | <0.001 | <0.001 |

**Method calculating body mass index**

We calculated body mass index using standardised major axis regression, a widely adopted method to calculate body mass index/body condition on mammals which was proposed by Peig & Green [2]. Standardised major axis regression of ln body mass against the natural log of tooth width (which is well correlated with body length) was calculated using the smatr package [3], described in detail in Thorley et al. [4]. One captive animal was removed from body mass index analysis because of missing data on tooth width.


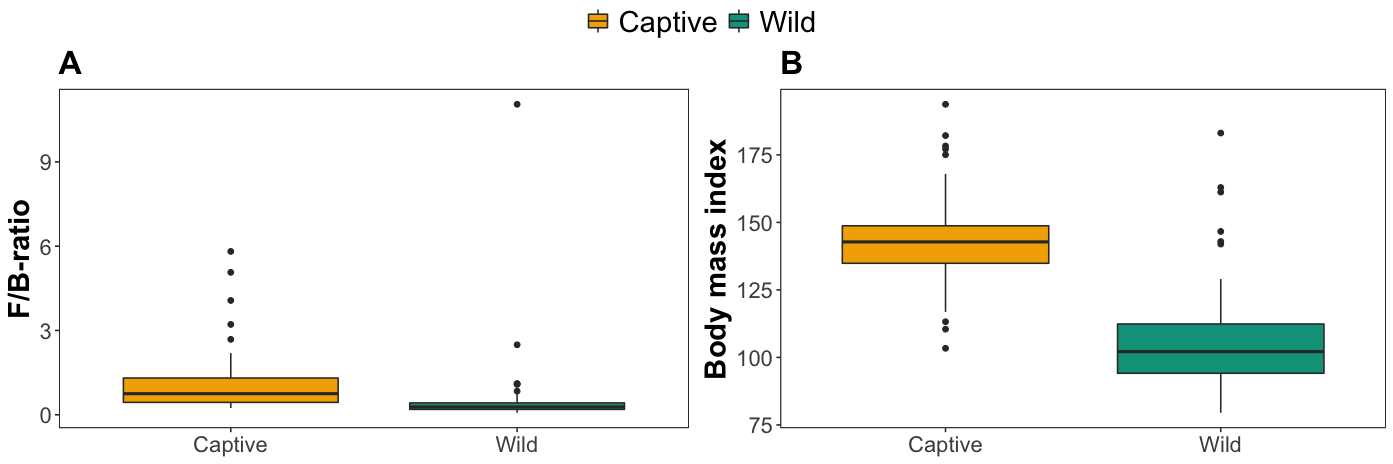


**Figure S3.** Differences in the ratio of relative abundances of Firmicutes/ Bacteroidetes and body mass index between captive and wild Damaraland mole-rats. A) Boxplot of ratio of relative abundances of Firmicutes/Bacteroidetes (F/B-ratio). One outlier from wild animal removed for visualization. B) Boxplot of body mass index (LM *p* < 0.001). Yellow boxes represent samples from captive individuals and blue-green boxes samples from wild individuals.


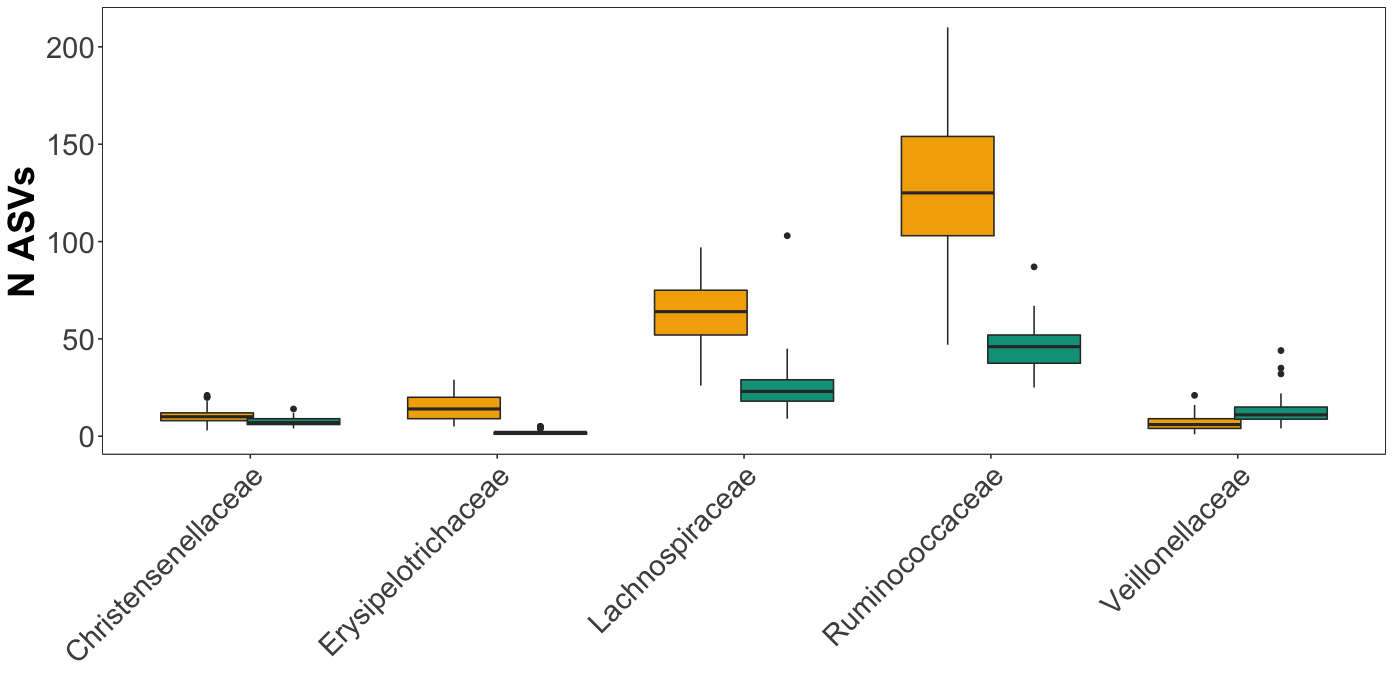


**Figure S4.** Boxplots of ASV richness of the five families within Firmicutes with the highest richness (mean richness > 10 ASVs) to explore what families explain increased richness of captive animals compared to wild.

**Table S7.** Percentage of ASVs classified to the taxonomic level for the full dataset separated by groups (wild and captive).

| **Group** | **domain** | **phylum** | **class** | **order** | **family** | **genus** | **species** |
| --- | --- | --- | --- | --- | --- | --- | --- |
| Captive | 100% | 99.7% | 99.6% | 98.8% | 91.6% | 57% | 8.93% |
| Wild | 100% | 98.1% | 97.8% | 96.7% | 88.8% | 44% | 8.04% |

**Table S8.** Meta-data for samples used in this study including BioSample numbers which can be found at short read archive (SRA) project PRJNA781121.

| **sample** | **Asample** | **SampleDate** | **Group** | **massindex** | **Plate_No** | **BioSample** |
| --- | --- | --- | --- | --- | --- | --- |
| 383 | A_383 | 2019-09-07 | Captive | 123.7 | 1 | SAMN23246719 |
| 382 | A_382 | 2019-09-07 | Captive | 148.23 | 2 | SAMN23246815 |
| 386 | A_386 | 2019-09-07 | Captive | NA | 3 | SAMN23246992 |
| 381 | A_381 | 2019-09-07 | Captive | 140.91 | 1 | SAMN23246735 |
| 380 | A_380 | 2019-09-07 | Captive | 145.55 | 1 | SAMN23246753 |
| 390 | A_390 | 2019-09-08 | Captive | 142.89 | 1 | SAMN23246722 |
| 391 | A_391 | 2019-09-08 | Captive | 142.65 | 1 | SAMN23246739 |
| 398 | A_398 | 2019-09-09 | Captive | 129.28 | 2 | SAMN23246813 |
| 399 | A_399 | 2019-09-09 | Captive | 138.26 | 1 | SAMN23246805 |
| 419 | A_419 | 2019-09-11 | Captive | 145.55 | 3 | SAMN23246975 |
| 426 | A_426 | 2019-09-11 | Captive | 137.68 | 2 | SAMN23246824 |
| 422 | A_422 | 2019-09-11 | Captive | 124.95 | 1 | SAMN23246800 |
| 424 | A_424 | 2019-09-11 | Captive | 178.3 | 3 | SAMN23246945 |
| 429 | A_429 | 2019-09-12 | Captive | 138.02 | 1 | SAMN23246749 |
| 432 | A_432 | 2019-09-13 | Captive | 175.06 | 2 | SAMN23246871 |
| 431 | A_431 | 2019-09-13 | Captive | 142.92 | 2 | SAMN23246851 |
| 430 | A_430 | 2019-09-13 | Captive | 133.95 | 3 | SAMN23246916 |
| 454 | A_454 | 2019-09-18 | Captive | 147.24 | 2 | SAMN23246864 |
| 459 | A_459 | 2019-09-20 | Captive | 141.68 | 2 | SAMN23246818 |
| 462 | A_462 | 2019-09-23 | Captive | 167.97 | 2 | SAMN23246810 |
| 474 | A_474 | 2019-09-24 | Captive | 177.15 | 3 | SAMN23246910 |
| 472 | A_472 | 2019-09-24 | Captive | 182.15 | 2 | SAMN23246855 |
| 480 | A_480 | 2019-09-25 | Captive | 137.27 | 1 | SAMN23246762 |
| 489 | A_489 | 2019-09-27 | Captive | 145.98 | 2 | SAMN23246892 |
| 492 | A_492 | 2019-09-29 | Captive | 167.77 | 3 | SAMN23246979 |
| 501 | A_501 | 2019-10-01 | Captive | 120.16 | 2 | SAMN23246840 |
| 511 | A_511 | 2019-10-03 | Captive | 139.81 | 2 | SAMN23246834 |
| 547 | A_547 | 2019-10-08 | Captive | 119.56 | 3 | SAMN23246928 |
| 555 | A_555 | 2019-10-09 | Captive | 135.15 | 2 | SAMN23246884 |
| 563 | A_563 | 2019-10-10 | Captive | 151.11 | 2 | SAMN23246860 |
| 565 | A_565 | 2019-10-15 | Captive | 127.7 | 2 | SAMN23246837 |
| 574 | A_574 | 2019-10-15 | Captive | 156.53 | 2 | SAMN23246842 |
| 576 | A_576 | 2019-10-15 | Captive | 133.67 | 2 | SAMN23246898 |
| 575 | A_575 | 2019-10-15 | Captive | 150.25 | 3 | SAMN23246977 |
| 588 | A_588 | 2019-10-16 | Captive | 145.29 | 2 | SAMN23246817 |
| 587 | A_587 | 2019-10-16 | Captive | 193.73 | 3 | SAMN23246932 |
| 613 | A_613 | 2019-10-19 | Captive | 120.21 | 3 | SAMN23246953 |
| 615 | A_615 | 2019-10-21 | Captive | 146.58 | 3 | SAMN23246961 |
| 617 | A_617 | 2019-10-23 | Captive | 145.64 | 2 | SAMN23246889 |
| 623 | A_623 | 2019-10-26 | Captive | 116.86 | 3 | SAMN23246966 |
| 664 | A_664 | 2019-11-05 | Captive | 113.21 | 3 | SAMN23246987 |
| 650 | A_650 | 2019-11-05 | Captive | 143.96 | 1 | SAMN23246760 |
| 653 | A_653 | 2019-11-05 | Captive | 163.87 | 2 | SAMN23246862 |
| 654 | A_654 | 2019-11-05 | Captive | 139.65 | 3 | SAMN23246941 |
| 652 | A_652 | 2019-11-05 | Captive | 135.52 | 1 | SAMN23246751 |
| 651 | A_651 | 2019-11-05 | Captive | 144.45 | 3 | SAMN23246988 |
| 655 | A_655 | 2019-11-05 | Captive | 110.46 | 2 | SAMN23246883 |
| 677 | A_677 | 2019-11-07 | Captive | 164 | 3 | SAMN23246912 |
| 676 | A_676 | 2019-11-07 | Captive | 142.38 | 1 | SAMN23246764 |
| 673 | A_673 | 2019-11-07 | Captive | 161.95 | 1 | SAMN23246754 |
| 672 | A_672 | 2019-11-07 | Captive | 135.25 | 1 | SAMN23246779 |
| 685 | A_685 | 2019-11-09 | Captive | 145.69 | 1 | SAMN23246720 |
| 686 | A_686 | 2019-11-09 | Captive | 103.38 | 2 | SAMN23246812 |
| 394 | A_394 | 2019-09-09 | Wild | 162.92 | 1 | SAMN23246713 |
| 549 | A_549 | 2019-10-08 | Wild | 104.32 | 1 | SAMN23246718 |
| 446 | A_446 | 2019-09-17 | Wild | 92.61 | 1 | SAMN23246724 |
| 442 | A_442 | 2019-09-17 | Wild | 93.64 | 1 | SAMN23246729 |
| 533 | A_533 | 2019-10-07 | Wild | 86.58 | 1 | SAMN23246731 |
| 428 | A_428 | 2019-09-12 | Wild | 94.08 | 1 | SAMN23246733 |
| 527 | A_527 | 2019-10-06 | Wild | 113.37 | 1 | SAMN23246744 |
| 495 | A_495 | 2019-09-30 | Wild | 118.8 | 1 | SAMN23246765 |
| 435 | A_435 | 2019-09-16 | Wild | 97.49 | 1 | SAMN23246770 |
| 598 | A_598 | 2019-10-17 | Wild | 112.05 | 1 | SAMN23246771 |
| 496 | A_496 | 2019-09-30 | Wild | 116.13 | 1 | SAMN23246776 |
| 456 | A_456 | 2019-09-19 | Wild | 91.41 | 1 | SAMN23246781 |
| 559 | A_559 | 2019-10-09 | Wild | 142.91 | 1 | SAMN23246786 |
| 580 | A_580 | 2019-10-15 | Wild | 146.65 | 1 | SAMN23246787 |
| 464 | A_464 | 2019-09-23 | Wild | 104.2 | 1 | SAMN23246788 |
| 449 | A_449 | 2019-09-17 | Wild | 94.64 | 1 | SAMN23246790 |
| 532 | A_532 | 2019-10-07 | Wild | 161.22 | 1 | SAMN23246791 |
| 498 | A_498 | 2019-10-01 | Wild | 99.36 | 1 | SAMN23246796 |
| 558 | A_558 | 2019-10-09 | Wild | 108.34 | 1 | SAMN23246799 |
| 393 | A_393 | 2019-09-09 | Wild | 94.84 | 1 | SAMN23246804 |
| 583 | A_583 | 2019-10-16 | Wild | 129.04 | 2 | SAMN23246809 |
| 450 | A_450 | 2019-09-17 | Wild | 121.4 | 2 | SAMN23246811 |
| 479 | A_479 | 2019-09-25 | Wild | 99.13 | 2 | SAMN23246814 |
| 522 | A_522 | 2019-10-06 | Wild | 86.01 | 2 | SAMN23246820 |
| 581 | A_581 | 2019-10-15 | Wild | 104.14 | 2 | SAMN23246831 |
| 461 | A_461 | 2019-09-23 | Wild | 104.23 | 2 | SAMN23246832 |
| 397 | A_397 | 2019-09-09 | Wild | 93.07 | 2 | SAMN23246833 |
| 516 | A_516 | 2019-10-06 | Wild | 98.25 | 2 | SAMN23246846 |
| 491 | A_491 | 2019-09-28 | Wild | 105.44 | 2 | SAMN23246859 |
| 395 | A_395 | 2019-09-09 | Wild | 110.86 | 2 | SAMN23246866 |
| 526 | A_526 | 2019-10-06 | Wild | 100.13 | 2 | SAMN23246868 |
| 405 | A_405 | 2019-09-10 | Wild | 99.71 | 2 | SAMN23246877 |
| 509 | A_509 | 2019-10-02 | Wild | 96.35 | 2 | SAMN23246880 |
| 556 | A_556 | 2019-10-09 | Wild | 84.31 | 2 | SAMN23246881 |
| 562 | A_562 | 2019-10-10 | Wild | 106.32 | 2 | SAMN23246885 |
| 542 | A_542 | 2019-10-07 | Wild | 183.09 | 2 | SAMN23246896 |
| 407 | A_407 | 2019-09-10 | Wild | 79.52 | 2 | SAMN23246901 |
| 494 | A_494 | 2019-09-30 | Wild | 116.62 | 3 | SAMN23246907 |
| 418 | A_418 | 2019-09-10 | Wild | 89.59 | 3 | SAMN23246918 |
| 473 | A_473 | 2019-09-24 | Wild | 96.44 | 3 | SAMN23246919 |
| 406 | A_406 | 2019-09-10 | Wild | 96.32 | 3 | SAMN23246923 |
| 476 | A_476 | 2019-09-25 | Wild | 141.97 | 3 | SAMN23246931 |
| 513 | A_513 | 2019-10-06 | Wild | 90.98 | 3 | SAMN23246936 |
| 433 | A_433 | 2019-09-16 | Wild | 94.14 | 3 | SAMN23246938 |
| 510 | A_510 | 2019-10-03 | Wild | 119.19 | 3 | SAMN23246942 |
| 443 | A_443 | 2019-09-17 | Wild | 96.91 | 3 | SAMN23246943 |
| 448 | A_448 | 2019-09-17 | Wild | 108.05 | 3 | SAMN23246948 |
| 584 | A_584 | 2019-10-16 | Wild | 99.24 | 3 | SAMN23246954 |
| 434 | A_434 | 2019-09-16 | Wild | 91.45 | 3 | SAMN23246955 |
| 469 | A_469 | 2019-09-24 | Wild | 111.78 | 3 | SAMN23246959 |
| 436 | A_436 | 2019-09-16 | Wild | 109.62 | 3 | SAMN23246965 |
| 447 | A_447 | 2019-09-17 | Wild | 86.39 | 3 | SAMN23246971 |
| 455 | A_455 | 2019-09-19 | Wild | 91 | 3 | SAMN23246982 |
| 460 | A_460 | 2019-09-21 | Wild | 106.99 | 3 | SAMN23246991 |
| 440 | A_440 | 2019-09-16 | Wild | 114.77 | 3 | SAMN23246994 |
| 457 | A_457 | 2019-09-20 | Wild | 105.9 | 3 | SAMN23246996 |

**References**

1. Willis A, Bunge J, Whitman T. Improved detection of changes in species richness in high diversity microbial communities. J R Stat Soc Ser C Appl Stat. 2016;963–77.

2. Peig J, Green AJ. New perspectives for estimating body condition from mass/length data: The scaled mass index as an alternative method. Oikos. 2009;118:1883–91.

3. Warton DI, Duursma RA, Falster DS, Taskinen S. smatr 3- an R package for estimation and inference about allometric lines. Methods Ecol Evol. 2012;3:257–9.

4. Thorley J, Bensch HM, Finn K, Clutton-Brock TH, Zöttl M. Fitness of breeders in social Damaraland mole-rats is independent of group size. bioRxiv. 2021;
